# Immigrant background and socioeconomic status are associated with severe COVID-19 requiring intensive care

**DOI:** 10.1038/s41598-022-15884-2

**Published:** 2022-07-15

**Authors:** Per Nordberg, Martin Jonsson, Jacob Hollenberg, Mattias Ringh, Ritva Kiiski Berggren, Robin Hofmann, Per Svensson

**Affiliations:** 1 Department of Clinical Science and Education, Södersjukhuset, Karolinska Institutet, Stockholm, Sweden; 2 Department of Anaesthesia, Intensive Care and Perioperative Services, Umea University Hospital, Umea, Sweden; 3 Center for Resuscitation Science, Karolinska Institutet, Stockholm, Sweden; 4 Function Perioperative Medicine and Intensive Care, Karolinska University Hospital, Stockholm, Sweden

**Keywords:** Diseases, Medical research, Risk factors

## Abstract

To determine whether immigrant background and socioeconomic status were associated with increased risk to develop severe Coronavirus disease 2019 (COVID-19) requiring mechanical ventilation at the intensive care unit and to study their effects on 90-day mortality. Nationwide case–control study with personal-level data from the Swedish Intensive Care register linked with socioeconomic data from Statistics Sweden and comorbidity data from the national patient register. For each case of COVID-19 treated with mechanical ventilation at the intensive care unit (outcome), 10 population controls were matched for age, sex and area of residence. Logistic and Cox regression were used to study the association between the exposure (immigrant background, income and educational level) and 90-day mortality. In total, 4 921 cases and 49 210 controls were matched. In the adjusted model, the risk of severe COVID-19 was highest in individuals born in Asia (Odds ratio [OR] = 2.44, 95% confidence interval [CI] = 2.20–2.69), South America (OR = 2.34, 95% CI = 1.82–2.98) and Africa (OR = 2.11, 95% CI = 1.76–2.50). Post-secondary education was associated with a lower risk of severe COVID-19 (OR = 0.75, CI = 0.69–0.82) as was the highest (vs. lowest) income quintile (OR = 0.87, CI = 0.77–0.97). In the fully adjusted Cox-regression analysis birth region of Africa (OR 1.38, CI = 1.03–1.86) and high income (OR 0.75, CI 0.63–0.89) were associated with 90-day mortality. Immigrant background, educational level and income were independently associated with acquiring severe COVID-19 with need for mechanical ventilation.

## Introduction

During the course of the pandemic, it has become increasingly clear that people from certain ethnical minorities have been more severely affected by Coronavirus Disease 2019 (COVID-19), which implies a multiplied risk for a more serious course of disease, need for hospitalization including intensive care treatment and, ultimately, death^1,2^. Although these disparities were early described^3^, they are still not sufficiently explained nor scientifically investigated^4^. The underlying causes are most likely multifactorial^4^, and may involve factors linked with socioeconomic status (SES) that in turn are linked with increased risk for cardiovascular disease^5–7^, and/or cardiovascular risk factors such as hypertension, diabetes and obesity, that are also strongly associated with severe COVID-19^8–11^.

To this point, existing data are incomplete on whether immigrant background per se, other socioeconomic factors, such as income and educational level, or cardiometabolic comorbidities independently increases the risk for severe disease with need of intensive care and on how they interact to result in disease. A systematic review on the literature of COVID-19 outcomes in migrant populations concluded that although migrants in high income countries are at high risk of exposure and infection with COVID-19, there is a need for high quality data on clinical outcomes in this group^12^. The differential risk may to some extent, but not fully, be accounted for by SES, living in urban areas or by higher burden of comorbidities^4^. Thus, all these factors need to be accounted for in the study design to be able to draw robust conclusions. Further it is also unclear whether immigrant background or SES have an impact on mortality also after admission to hospital.

Therefore, in this nationwide case–control study, we hypothesized that immigrant background in Sweden and SES were associated with a higher risk for acquiring severe COVID-19 with need for mechanical ventilation at the intensive care unit (ICU) independently of cardiometabolic comorbidities. Utilizing comprehensive Swedish national registers with high quality individual data from the intensive care treatment, comorbidity, socioeconomic factors and migration, the primary aim of this study was to investigate whether immigrant background in Sweden and socioeconomic status were associated with a higher risk to develop severe COVID-19 requiring mechanical ventilation at the intensive care unit (ICU). As a secondary aim, we assessed whether the patients admitted the ICU with immigrant background had increased 90-day mortality compared to Swedish-borne patients, also taking into account socioeconomic and clinical factors.

## Methods

### Study design and ethics

This is a nationwide case–control study, based on data from the Swedish Intensive Care Register (SIR) on patients (cases) with COVID-19 admitted to the ICU. The study period for admission to ICU was 1st of March 2020 until 15th of June 2021. For each case, 10 controls were randomly selected from the Swedish Population Register and matched by age, sex and area of residence. The study data base was thereafter merged with multiple mandatory Swedish national registries at the National Board of Health and Welfare using each individual’s unique personal identification number^13–15^.

The study was approved by the Swedish Ethical Review Authority (identification number 2020/124-31/4). The Swedish Ethical Review Authority provided the waiver for obtaining informed consent, due to the nature of this register study. The study used pseudonymized data and involved minimal infringement of personal integrity which is in adherence to the guidelines of the Swedish Ethical Review Authority.

### Setting and patients

Sweden has 10.4 million inhabitants and many regions have been struck hard by the COVID-19 epidemic. Approximately 20% of the Swedish population is born abroad (Statistics Sweden, https://www.scb.se/en/) with the following distribution of region of births from different parts of the world; Nordic countries (2.3%), Europe (6.5%), Africa (2.2%), South America (0.7%), Asia (7.6%), other (0.5%). The Swedish health care is tax funded, all acute treatments including admission to the ICU, are based on medical decisions and do not involve private-economic considerations, thereby reducing unequal access to health care. This study included consecutive patients with severe COVID-19, defined in this study as admitted to the ICU and requiring mechanical ventilation. All eligible patients during the study period between 1st of March until 11th of May 2020 were included as cases in the study.

### National registries and data collection

SIR, the primary data source, is a national register with a 95% coverage of all ICU admissions in Sweden. It contains individual data regarding clinical presentation at admission such as Simplified Acute Physiologic Score (SAPS), and procedures and treatments performed during the ICU stay, e.g. mechanical ventilation.

After selecting the study patients, the National Patient Register^16^ was used to collect primary or secondary diagnoses from previous hospital admissions and outpatients’ visits coded according to the International Classification of Diseases (ICD) version 10 within the 15 years preceding the admission. The Prescribed Drugs Register contains information on all dispensed drugs according to the Anatomical Therapeutic Chemical Classification (ATC). We collected individual data on dispensed drugs prescribed and claimed within six months before the study period. As the last step, these individual data were linked with data from Statistics Sweden which includes annual measurements on several socioeconomic and demographic factors, including income, education and region of birth.

### Definition of exposures

#### Immigrant background

Immigrant background was defined as place of birth in a region outside Sweden and categorized according to the definition supplied by Statistics Sweden; Nordic countries, Europe, Asia, Africa and South America. North America, Oceania and former Soviet Union were categorized as “other” due to few cases of severe COVID-19.

#### Income

Income was defined as the yearly household disposable income. Men and women were categorized into quintiles separately based on their income during the last 5 years.

#### Education

Education was defined as the highest level of completed education. The educational level was categorized into primary education, secondary education, and post-secondary education.

### Outcomes

The primary outcome (all cases) was defined as an ICU admission due to COVID-19 (with a laboratory confirmed Sars-Cov2 infection), registered in SIR, with at least one episode of invasive mechanical ventilation during the ICU stay. The secondary outcome was all-cause mortality at 90-days among the cases admitted to the intensive care.

### Definition of covariates

Hypertension (defined as previous diagnosis of ICD I10 or prescription of antihypertensive drugs as described previously^17^), hyperlipidemia (ICD E78 or prescription of lipid lowering drugs), diabetes mellitus type 2(ICD E11 or prescription of antidiabetic drugs), diabetes mellitus type 1 (ICD E10), obesity (ICD E66), heart failure (ICD I50.1, I50.9), atrial fibrillation (ICD I48), asthma (ICD J45), chronic obstructive pulmonary disease (ICD J44), chronic kidney disease (ICD N18), and malignancy (ICD C, D40-48) were included. A history of cardiovascular disease (CVD) was defined as a record of either MI (ICD I21, I22), ischemic heart disease (ICD I25), ischemic stroke (ICD I63), or peripheral vascular disease (ICD I70-I73), in the Swedish Patient Register.

### Statistical methods

Baseline characteristics for categorial variables are presented as frequencies and proportions while continuous variables are presented as medians and quartiles. Each case of severe COVID-19 requiring mechanical ventilation were matched with 10 population-based controls matched on age, sex and area of residence. The case–control data were analyzed with logistic regression. The results are presented as crude results (adjusted for age and sex), mutually adjusted results (adjusted for age, sex and exposures) and fully adjusted results (age, sex, exposures, civil status, hypertension, hyperlipemia, diabetes type 1, diabetes type 2, obesity, heart failure, atrial fibrillation, asthma, chronic obstructive pulmonary disease, chronic kidney disease, malignancy and cardiovascular disease). Additional analyses were performed by cases of severe COVID-19 and 90-day survival using Cox regression. All results from the regression analyses were presented as odds ratios (OR) or hazard ratios (HR) with 95% confidence intervals. The statistical calculations were performed using R version 4.0.3 (R Foundation for Statistical Computing 2020, Vienna, Austria).

### Ethical approval

Identification number 2020/124-31/4.

## Results

A total of 4.921 cases of severe COVID-19 requiring mechanical ventilation at the ICU and 49.210 controls were included in the study. The baseline characteristics are presented in Table 1. Cases diagnosed with severe COVID-19 had lower median income, a higher proportion with only primary education and were to a larger extent born outside Europe compared to the matched control subjects. They also had a higher prevalence of hypertension, type 2 diabetes and obesity. The baseline characteristics by region of birth are presented in Table 2. Cases with a region of birth outside Europe were in general younger and had a lower income compared to cases with a region of birth in Europe. A similar pattern of comorbidities was observed across the different regions of birth but type 2 diabetes was more common in cases with a region of birth in Africa. The SAPS score and vital parameters at admission to ICU were largely similar in the different groups.

**Table 1:** Baseline characteristics and outcome of cases and population controls.

	Controls	Cases	SMD
n	49,210	4921	
Age (median [IQR])	64 [55, 72]	64 [55, 72]	< 0.001
Female Sex (%)	14,470 (29.4)	1447 (29.4)	< 0.001
**Region of birth (%)**			0.345
Sweden	37,224 (75.7)	2989 (60.8)	
Nordic countries	1669 (3.4)	202 (4.1)	
Europe	4092 (8.3)	534 (10.9)	
Asia	4286 (8.7)	861 (17.5)	
Africa	1199 (2.4)	213 (4.3)	
South America	495 (1.0)	94 (1.9)	
Other	239 (0.5)	23 (0.5)	
**Income quintile (%)**			0.279
Q1	9377 (19.1)	1429 (29.1)	
Q2	9737 (19.8)	1069 (21.8)	
Q3	9899 (20.2)	906 (18.5)	
Q4	10,004 (20.4)	801 (16.3)	
Q5	10,101 (20.6)	704 (14.3)	
Education (%)			0.212
Primary	10,216 (21.1)	1385 (29.1)	
Secondary	22,023 (45.5)	2141 (45.0)	
Post-Secondary	16,196 (33.4)	1236 (26.0)	
**Comorbidities** (%)			
Hypertension	11,589 (23.6)	2829 (57.5)	0.737
Diabetes1	967 (2.0)	204 (4.1)	0.127
Diabetes2	4255 (8.6)	1200 (24.4)	0.434
Cardiovascular disease	5012 (10.2)	763 (15.5)	0.219
Atrial fibrillation	3247 (6.6)	570 (11.6)	0.174
Chronic kidney disease	795 (1.6)	291 (5.9)	0.227
Heart failure	1644 (3.3)	383 (7.8)	0.195
Asthma	1807 (3.7)	566 (11.5)	0.299
Malignancy	4175 (8.5)	440 (8.9)	0.016
Obesity	1781 (3.6)	894 (18.2)	0.480
COPD	1287 (2.6)	299 (6.1)	0.170
**Survival** (%)			
7 days	49,194 (100.0)	4534 (92.1)	0.411
30 days	49,158 (99.9)	3624 (73.6)	0.840
90 days	48,652 (98.9)	3361 (68.3)	0.906

*SMD* standard mean deviation; *Q* quintile; *COPD* chronic obstructive pulmonary disease.

**Table 2:** Patient characteristics, intensive care measures and outcomes among patients admitted to ICU with severe Covid-19.

	Sweden	Nordic countries	Europe	Asia	Africa	South America	Other	SMD
n	2989	202	534	861	213	94	23	
Age (median [IQR])	66 [56, 74]	69 [61, 74]	65 [56, 72]	59 [50, 67]	57 [48, 67]	64.0 [55, 71]	61.0 [54, 66]	0.392
Sex = Female (%)	878 (29.4)	84 (41.6)	137 (25.7)	247 (28.7)	66 (31.0)	28 (29.8)	6 (26.1)	0.120
**Income** (%)								0.612
Q1	494 (16.5)	54 (26.7)	192 (36.4)	516 (60.0)	134 (64.1)	28 (29.8)	7 (30.4)	
Q2	689 (23.1)	38 (18.8)	135 (25.6)	145 (16.9)	33 (15.8)	20 (21.3)	9 (39.1)	
Q3	619 (20.7)	45 (22.3)	89 (16.9)	100 (11.6)	28 (13.4)	22 (23.4)	3 (13.0)	
Q4	608 (20.3)	32 (15.8)	67 (12.7)	60 ( 7.0)	14 ( 6.7)	18 (19.1)	2 ( 8.7)	
Q5	579 (19.4)	33 (16.3)	45 ( 8.5)	39 ( 4.5)	0 ( 0.0)	6 ( 6.4)	2 ( 8.7)	
**Education** (%)								0.329
Primary	776 (26.3)	57 (28.5)	178 (35.1)	279 (35.1)	64 (34.6)	21 (22.8)	8 (34.8)	
Secondary	1470 (49.7)	102 (51.0)	216 (42.6)	245 (30.8)	66 (35.7)	37 (40.2)	5 (21.7)	
Post-Secondary	710 (24.0)	41 (20.5)	113 (22.3)	272 (34.2)	55 (29.7)	34 (37.0)	10 (43.5)	
**Comorbidities** (%)								
Hypertension	1825 (61.1)	136 (67.3)	328 (61.4)	386 (44.8)	92 (43.2)	50 (53.2)	11 (47.8)	0.231
Type 1 diabetes	136 ( 4.6)	5 ( 2.5)	29 ( 5.4)	20 ( 2.3)	11 ( 5.2)	2 ( 2.1)	1 ( 4.3)	0.089
Type 2 diabetes	696 (23.3)	52 (25.7)	158 (29.6)	190 (22.1)	72 (33.8)	24 (25.5)	7 (30.4)	0.115
Cardiovascular disease	476 (15.9)	38 (18.8)	110 (20.6)	107 (12.4)	14 ( 6.6)	14 (14.9)	4 (17.4)	0.160
Atrial fibrillation	422 (14.1)	31 (15.3)	62 (11.6)	43 ( 5.0)	7 ( 3.3)	4 ( 4.3)	0 ( 0.0)	0.286
Chronic kidney disease	185 ( 6.2)	7 ( 3.5)	34 ( 6.4)	47 ( 5.5)	13 ( 6.1)	4 ( 4.3)	1 ( 4.3)	0.063
Heart failure	247 ( 8.3)	26 (12.9)	55 (10.3)	45 ( 5.2)	7 ( 3.3)	3 ( 3.2)	0 ( 0.0)	0.243
Asthma	364 (12.2)	26 (12.9)	54 (10.1)	80 ( 9.3)	20 ( 9.4)	17 (18.1)	5 (21.7)	0.157
Malignancy	300 (10.0)	19 ( 9.4)	54 (10.1)	44 ( 5.1)	13 ( 6.1)	9 ( 9.6)	1 ( 4.3)	0.112
Obesity	575 (19.2)	44 (21.8)	105 (19.7)	112 (13.0)	32 (15.0)	23 (24.5)	3 (13.0)	0.141
COPD	202 ( 6.8)	18 ( 8.9)	41 ( 7.7)	27 ( 3.1)	3 ( 1.4)	5 ( 5.3)	3 (13.0)	0.193
**At ICU admission,** (mean (SD))								
SAPS 3	59.3 (11.1)	59.0 (9.8)	57.2 (10.4)	54.9 (8.9)	56.4 (9.7)	57.8 (9.1)	57.3 (8.9)	0.186
Temperature, °C	37.6 (1.2)	37.5 (1.0)	37.6 (1.2)	37.7 (1.0)	37.6 (1.0)	37.6 (1.0)	37.6 (0.9)	0.082
Heart rate, beats/min	95.5 (23.6)	91.5 (23.7)	94.9 (22.5)	93.9 (20.1)	98.8 (21.4)	96.1 (20.2)	89.6 (16.0)	0.177
Systolic blood pressure, mmHg	119.6 (30.2)	123.4 (31.8)	122.8 (29.6)	119.4 (26.8)	123.6 (31.5)	119.3 (26.9)	114.0 (20.4)	0.142
pH	7.4 (0.1)	7.4 (0.1)	7.4 (0.1)	7.4 (0.1)	7.4 (0.1)	7.4 (0.1)	7.4 (0.1)	0.093
FiO2, %	74.0 (19.0)	72.8 (19.6)	75.5 (18.7)	72.4 (18.6)	72.3 (19.7)	81.8 (18.0)	69.3 (19.8)	0.229
PaO2, kPa	10.0 (6.4)	9.9 (6.2)	9.4 (4.0)	9.9 (5.9)	9.3 (2.7)	9.9 (9.4)	12.4 (13.5)	0.119
**Survival,** number (%)								
7 days	2705 (90.5)	190 (94.1)	499 (93.4)	825 (95.8)	199 (93.4)	90 (95.7)	21 (91.3)	0.098
30 days	2150 (71.9)	146 (72.3)	403 (75.5)	681 (79.1)	156 (73.2)	70 (74.5)	15 (65.2)	0.109
90 days	1999 (66.9)	135 (66.8)	370 (69.3)	627 (72.8)	149 (70.0)	65 (69.1)	14 (60.9)	0.091

Stratified by region of birth.

*SMD* standard mean deviation; *Q* quintile; *COPD* chronic obstructive pulmonary disease; *SAPS* Simplified acute physiology score.

### Primary outcome

**Figure 1:**
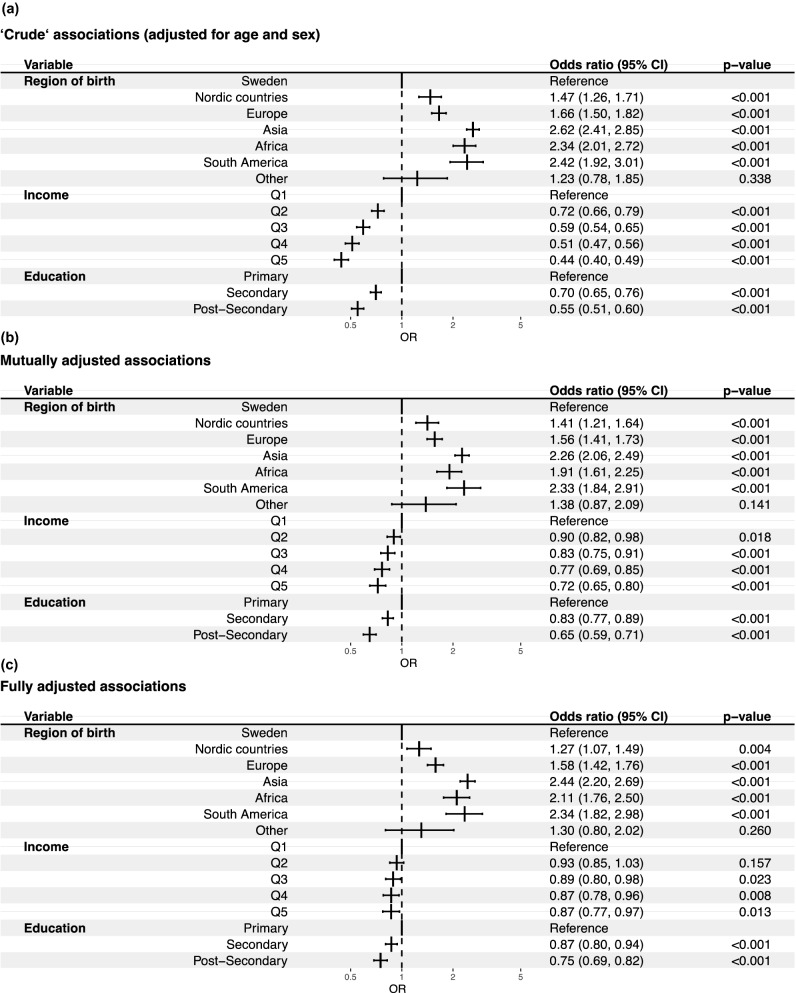
(**a**) Associations between region of birth, income and education with severe COVID-19. Logistic regression analyses. Crude = adjusted for age and sex. (**b**) Mutually adjusted = adjusted for region of birth, income, education age and sex. (**c**) Fully adjusted = Mutually adjusted + hypertension, diabetes (1 + 2), cardiovascular disease, atrial fibrillation, chronic kidney disease, heart failure, asthma, malignancy, obesity, COPD.

The results from the logistic regression adjusted for age and sex are shown in Fig. 1A. There was a clear association between region of birth and severe COVID-19 requiring invasive ventilation at the ICU. The highest risk was seen among persons from Asia (OR = 2.62, 95% CI = 2.41–2.85) followed by South America (OR = 2.41, 1.92–3.01) and Africa (2.34, 95% CI = 2.01–2.72). A higher income was associated with a lower risk for severe COVID-19, the highest (vs. lowest) income quintile (OR = 0.44, 95% CI = 0.40–0.49). A similar association was seen for education with a lower risk associated with a higher level of education. When mutually adjusted (Fig. 1B), similar associations with the outcome were observed for region of birth and education, but not for income where no clear association was seen.

After further adjustment for comorbidities (Fig. 1C), the results remained similar with a strong association of region of birth, especially Asia (OR 2.44, 95% CI = 2.20–2.69), South America (OR = 2.34, 95% CI = 1.82–2.98) and Africa (OR = 2.11, 95% CI = 1.76–2.50), weaker association for educational level (OR = 0.75, 95% CI = 0.0.69–0.82) and for income (OR 0.87, 95% CI = 0.77–0.97 with the outcome.

### Secondary outcome

**Figure 2:**
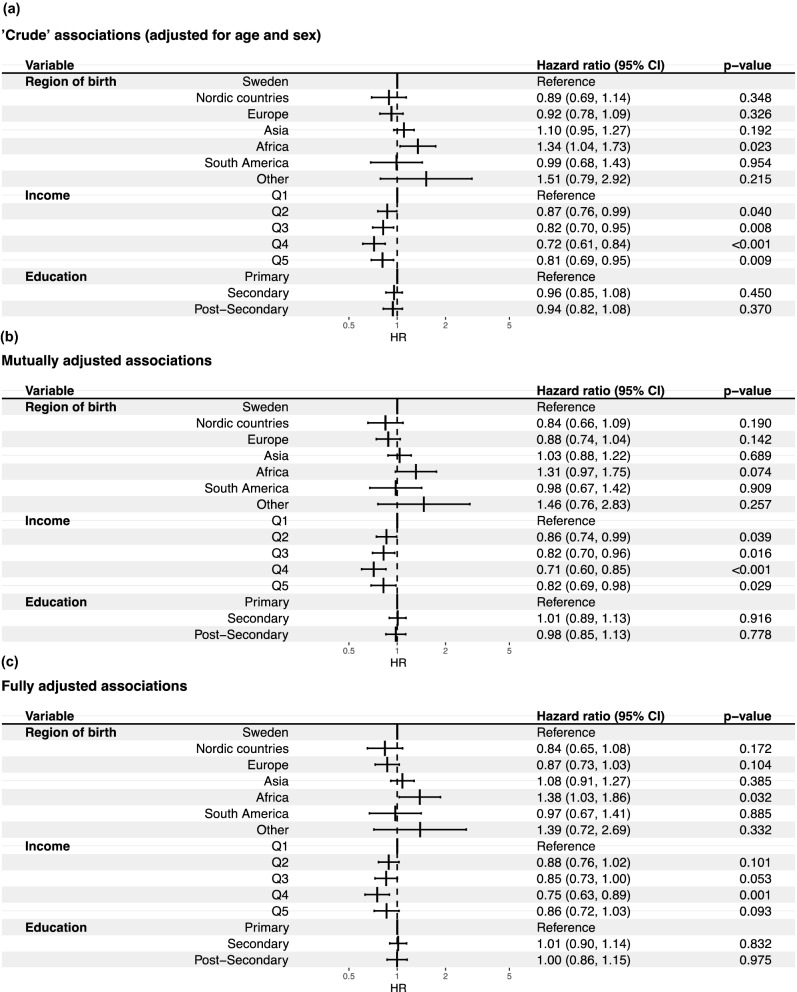
(**a**) Cox regression analyses on 90-day survival. Crude = adjusted for age and sex. (**b**) Mutually adjusted = adjusted for region of birth, income, education age and sex. (**c**) Fully adjusted = Mutually adjusted + hypertension, diabetes (1 + 2), cardiovascular disease, atrial fibrillation, chronic kidney disease, heart failure, asthma, malignancy, obesity, COPD.

At the 90-day follow up, a total of 1560 patients out of 4921 admitted to ICU with invasive ventilation had died. In Fig. 2 the results from the Cox regression are shown. In contrast to the associations between immigrant background and severe disease observed in the case–control analyses, the only significant association was observed between immigrant background from the African region and 90-day mortality among patients admitted to the ICU. When compared to patients with severe COVID-19 born in Sweden, the HR in the fully adjusted model for patients born in Africa was 1.38 (95% CI = 1.03–1.86), patients born in Asia 1.08 (95% CI = 0.91–1.27) and patients born in South America 0.97 (95% CI = 0.67–1.41). Moreover, only a weak association with income, and no association of 90-day mortality with educational level was seen in the fully adjusted model.

**Figure 3:**
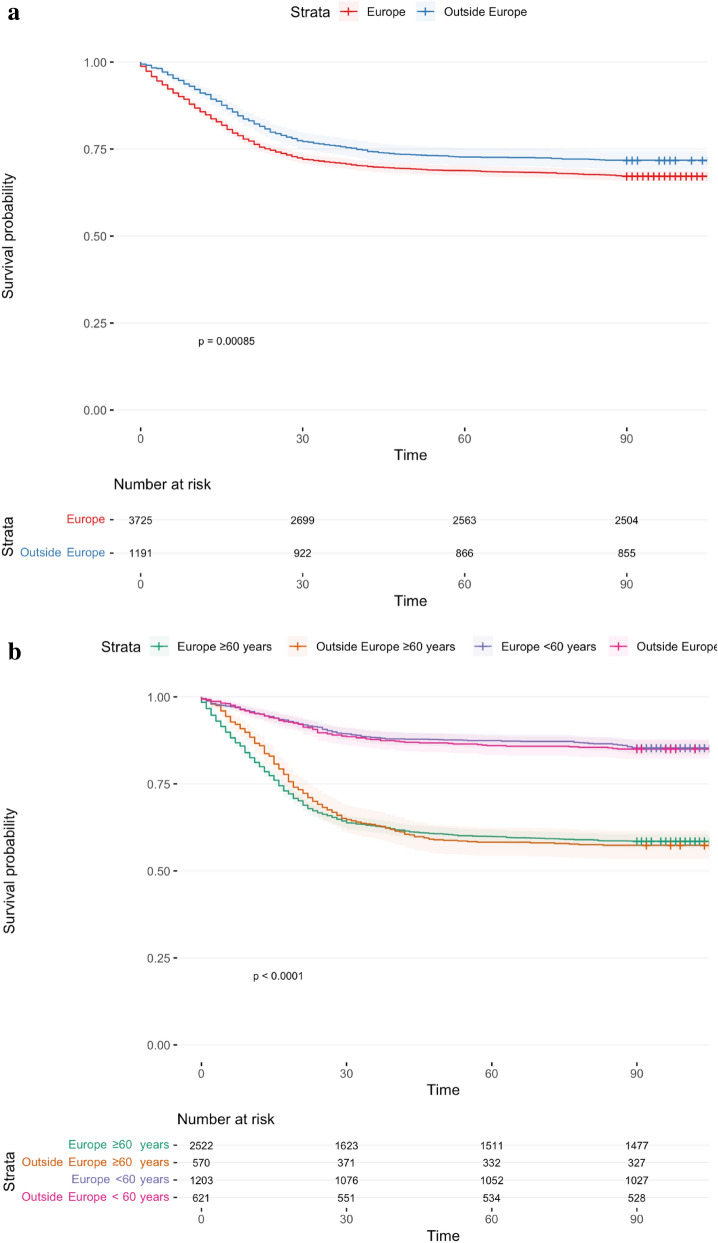
(**a**) Kaplan–Meier survival curves for patients with severe Covid-19 stratified by region of birth (Europe vs. outside Europe). (**b**) Kaplan–Meier survival curves for patients with severe Covid-19 stratified by age (younger than 60 years vs. older than/or equal to 60 years) and region of birth (Europe vs outside Europe).

In Fig. 3a,b, the patient’s cohort is divided into those born within the European union (EU) and those born outside the EU and by age-group. The Kaplan Meier curves show the probability of survival to 90 days in these two groups (3a) and with a stratification by age (< 60 years of age vs.  ≥ 60 years of age) in 3b. There are some minor differences within the two age groups, but significant. Figure 3b clearly shows the importance of age as a major risk factor for 90-day mortality in the two groups.

### Discussion

The COVID-19 pandemic has clearly demonstrated the importance of social determinants of health such as ethnicity/minority status, socioeconomic status for health outcomes, with a more profound impact in low socioeconomic groups. In this study, we determined the association of immigrant background and SES with the most critical COVID-19 outcomes, i.e. patients requiring mechanical ventilation at the ICU and death, in relation to comorbidities. The main finding was that an immigrant background, especially with origin in the regions of Africa, South America and Asia, was independently associated with an increased risk to develop severe COVID-19 when adjusting for socioeconomic factors and comorbidity. In contrast, both a higher level of education and a higher income were associated with decreased risk for a severe course of disease. However, once admitted to the ICU with need of mechanical ventilation, only in individuals born in Africa an association with increased 90-day mortality was observed, whereas no such association was seen with educational level, and only a weak association with income.

There are several potential explanations for the increased risk of severe COVID-19 in individuals with immigrant background. First, as the determinants of disease severity appear to mostly depend on host factors^18^, genetic predisposition for acquiring a more severe course of disease is likely. Several genetic factors have been proposed to increase the risk of COVID-19^19,20^. However, as our data is limited with regard to genetic information, we cannot include these factors in our assessment in the current study. Second, as cultural and social interactions differ between different societal groups, cluster outbreaks may occur in communities with a high proportion of individuals with certain backgrounds. In addition, health seeking patterns may differ in certain population groups which may imply patient delay and leading to deterioration prior to hospital arrival^21^. Third, systemic factors, such as equity within the health care system with regard to patient/doctor ratio and access to nearby health facilities may be important^22,23^. Although Swedish health care is tax funded there may be structural inequities for people with different background. Fourth, people with lower educational level may neither be able to afford nor have the possibility to work from home, and therefore expose themselves to a higher risk to get infected^24^. A lower level of education may also be linked to low health literacy implying a lower adherence to the recommendations given by public health authorities^25^. Another plausible explanations may be that chronic diseases important for this association are more prevalent in lower educational groups which has been shown for diabetes, obesity and hypertension^26,27^. In the current analysis, despite the fact that some of these conditions were overrepresented in certain immigrant groups, our findings clearly show that the exaggerated risk associated with immigrant background was independent of prior disease and risk factor burden.The attenuation of the observed association for income with severe COVID-19 in the fully adjusted analysis indicate that such socioeconomic variables are important and may be important mediators for the association between region of birth and severe COVID-19.

Our result confirm and extend case–control data from the UK where people from different ethnical minorities had a higher risk of hospital admission^28^. Their findings were independent of community deprivation and comorbidities whereas we matched for community but also adjusted for individual level socioeconomic data.

To compare measures and outcome in patients with severe COVID-19 is a challenge as the definition of severe disease and selection of study population vary substantially. In our study, several high-quality national registries have been used to achieve a well-characterised, homogenous, population with severe COVID-19 patients, all requiring mechanical ventilation at the ICU. The standard of care is most likely similar across the country since all the ICU’s are publicly operated and follow national recommendations. We did only see minor outcome differences in 90-day survival in the adjusted model for birth region of Africa and weak association with income. Our results are almost similar to Zakeri et al.^28^ which could not identify any association between immigrant background and the risk of mortality in the survival analysis. This is also in line with results from the US where no significant associations were seen after adjustment for covariates^29^. In this ICU population, about half of the patients had an immigrant background, most commonly from Asia. The discrepancy between increased risks for being admitted to ICU with severe COVID -19 but similar prognosis after ICU admission points to that the pandemic spread during the study period differed in the Swedish population with a higher spread in groups with immigrant background and in those with lower SES. We believe that the Swedish health care system is an appropriate context for assessing inequities since it is virtually fully tax funded and admission to the ICU including necessary measures, is based on medical decisions only and does not involve private-economic considerations.

In summary, we have tried to address the concerns about the lack of collection of high quality socioeconomic and data on region of birth to assess risk and outcome during the COVID-19 pandemic^30^. Our findings, originating from high-quality national registries in Sweden which has been experiencing a serious epidemic with high incidence and mortality rates, may have several implications; to strengthen measures to prevent transmission of COVID-19 in vulnerable populations; and to take into account socioeconomic factors and immigrant background in the prioritization of vaccinations among individuals at highest risk to develop severe disease.

The authors are aware of several limitations. The information on region of birth is limited to continents and by country or regions, which may affect the results as the heterogenicity of the populations may differ in different regions. We did not have any information concerning the rate of mild infection with COVID-19 among control subjects although none of the controls were admitted to hospital during the study period. Conditions primarily managed in primary health care or previously undiagnosed, such as obesity, may have been underestimated in the control population. Finally, the observational design of the study cannot exclude the potential of residual confounding and the results should be interpreted as such.

### Conclusion

Immigrant background, educational level and income were independently associated with acquiring severe COVID-19 with need for mechanical ventilation.

## Data Availability

Data are available upon reasonable request. The data underlying this article cannot be shared publicly due to the privacy of individuals that participated in the study. The data will be shared on reasonable request to the corresponding author.
